# A retrospective comparative study of mortality and causes of death among patients with metal-on-metal and metal-on-polyethylene total hip prostheses in primary osteoarthritis after a long-term follow-up

**DOI:** 10.1186/1471-2474-11-78

**Published:** 2010-04-23

**Authors:** Tuomo Visuri, Håkan Borg, Pekka Pulkkinen, Pekka Paavolainen, Eero Pukkala

**Affiliations:** 1 Research Institute of Military Medicine, Helsinki, Finland; 2 Clinics for Orthopaedics and Traumatology, Central University Hospital, Helsinki, Finland; 3 Department of Public Health, University of Helsinki, Helsinki, Finland; 4 Orton Orthopaedic Hospital, Helsinki, Finland; 5 Finnish Cancer Registry, Helsinki, Finland, and School of Public Health, University of Tampere, Tampere, Finland

## Abstract

**Background:**

All patients with total hip arthroplasty (THA), especially those with metal-on-metal (MM) THA, are exposed to metallic particles and ions, which may cause total or site-specific mortality. We analyzed the causes of total and site-specific mortality among a cohort of patients with MM and with metal-on-polyethylene (MP) THA after a long follow-up time.

**Methods:**

Standardized mortality ratios (SMR) of total and site-specific causes of death were calculated for 579 patients with MM (McKee-Farrar) and 1585 patients with MP (Brunswik, Lubinus) THA for primary osteoarthritis.

**Results:**

Mean follow-up time was 17.9 years for patients with MM and 16.7 years for patients with MP. Overall SMR was 0.95 for the MM cohort and 0.90 for the MP cohort, as compared to the normal population. Both cohorts showed significantly decreased mortality for the first decade postoperatively, equal mortality over the next 10 years, and significantly increased mortality after 20 years. Patients with MM THA had higher cancer mortality (SMR 1.01) than those with MP THA (SMR 0.66) during the first 20 years postoperatively, but not thereafter.

**Conclusion:**

Both MM and MP prostheses are safe based on total and site-specific mortality of recipients during the first 20 postoperative years in comparison with the general population.

## Background

Metal-on-metal (MM) total hip arthroplasty (THA) and especially MM hip resurfacing techniques have recently regained popularity due to successful mid-term results in young patients [1-4]. Patients with these prostheses will be exposed to cobalt and chromium particles and ions for decades.

MM articulations give rise to particles that are typically 20 to 90 nm in size, with a mean size of approximately 50 nm [5-7]. Because of their small size, MM particles disseminate widely throughout the body, particularly to the lymph nodes, liver, spleen, and bone marrow [8-12]. The number of these metallic particles is estimated to be 1000 times greater than that from metal-on-polyethylene (MP) prostheses [13]. These particles have a large surface area, and their degradation increases the concentrations of cobalt and chromium ions in the body fluids. An autopsy performed more than 25 years after successful implantation of a McKee-Farrar prosthesis (Howmedica International, Limerick, Ireland) showed elevated levels of cobalt and chromium *in liver*, even in the absence of specific particulate debris in remote sites. This finding suggests that the metal is stored in the liver as organometallic complexes or in nanoparticulate form so small that it is beyond the resolution of electron microscopic analysis [11].

In an early study [14], nine patients with a McKee-Farrar prosthesis had an approximately 3-fold elevation of chromium and an 11-fold elevation of cobalt in the whole blood, and a 15-fold elevation in chromium in the urine, compared with the preoperative values. High serum metal levels were observed in eight McKee-Farrar patients after a 20-yearfollow-up, 9-fold higher for chromium and 3-fold higher for cobalt compared with controls [15]. Five patients with Ring prostheses (Downs Bros, Mitcham, United Kingdom) for an average of 33 years had approximately 5-fold greater serum cobalt levels and 3-fold greater serum chromium levels compared to patients with a primary or revised MP prostheses or patients with osteoarthritis (OA) and no implants [16]. Elevated serum chromium and cobalt levels have also been reported in patients with MP prostheses, but the concentrations are lower than those in patients with MM prostheses [17].

Both *in vivo *and *in vitro *studies demonstrate chromosomal aberrations and DNA damage in association with debris from cobalt chromium MM and MP prostheses. An increase in chromatid breaks and gaps are observed in the bone marrow adjacent to the worn implant [18]. Compared with patients at primary arthroplasty, patients with mixed types of cobalt chromium prostheses who underwent revision arthroplasty had a 2.5-fold increase in the number of peripheral blood lymphocytes in aneuploidy and a 3.5-fold increase in chromosomal translocations [19]. Debris from worn hip and knee prostheses damages chromosomes in a dose-dependent manner in human tissue culture, and is specific to the type of metal. Cobalt and chromium concentrations correlate with chromosomal breakage and aneuploidy events, and titanium concentrations correlate with aneuploidy events [20]. A 2-year prospective study in patients with MM hip prostheses revealed a significant increase in whole blood cobalt and chromium concentrations as well as in chromosome aneuploidy and translocations in the peripheral blood lymphocytes [21].

Therefore, patients with MM and MP hip prostheses are exposed to cobalt and chromium particles and ions, which are potentially genotoxic. Long-term exposure to these metals may affect mortality in these patients. We analysed the causes of death in patients with MM and MP total hip articulations over a long follow-up period.

## Methods

Basic patient material was identified from the medical records of two regional orthopaedic hospitals in Finland. When only patients diagnosed with OA were selected, the MM arthroplasty group comprised 579 patients who underwent a McKee-Farrar THA (all cobalt-chromium-molybdenum) between 1967 and 1973, and the MP series comprised 1585 patients who had either a Brunswik or Lubinus (Waldemar Link GmbH & Co., Hamburg, Germany) prosthesis (cobalt-chromium-molybdenum stem) and who were operated on between 1973 and 1985. Data for this patient population were reported previously [22]. The proportion of women in the MM group was 66% and that in the MP group was 61%.

The dates and causes of death during 1971-2005 for the cohort members were obtained from Statistics Finland by record linkage using a personal identifier code as the key. The coverage of the cause-of-death statistics is virtually complete.

Calculation of person-years at risk started from the operation and ended at death or closing-date of the study, December 31, 2005. The number of observed cases for each cause of death and person-years at follow-up was stratified by sex, calendar period, 5-year age group, and follow-up time since the operation. The expected number of patients to die from each cause was calculated by applying the number of person-years in each stratum to the corresponding mortality rate in a similar Finnish population. The calendar periods used were 1971-1974, 1975-1982, 1983-1990, 1991-1998, and 1999-2005; and the follow-up categories were < 2, 2-9, 10-19, and > 20 years since the operation. The standardized mortality ratio (SMR) was expressed as the ratio of observed and expected number of cases. The 95% confidence intervals (95% CI) were defined based on the assumption that the number of observed cases followed a Poisson distribution. The relative mortality risk between patients with MM or MP THA was calculated as the rate ratio (RR) of the two SMRs. The 95% confidence limits of the RR were calculated assuming Poisson distributions.

The list of causes of death included all 53 categories for which Statistics Finland routinely produces mortality rates based on the International Classification of Diseases, revisions 8, 9, and 10 [23].

This study was approved by Welfare and Health Administration of Finland (DNro 639/103/91)

## Results

By the end of 2005, 528 (91.1%) of the 579 patients with an MM prosthesis and 1299 (82.0%) of the 1585 patients with an MP prosthesis had died. The mean follow-up times were 17.9 and 16.7 years, respectively (Table 1). Both groups of patients had a total mortality rate slightly below the national average. All-cause mortality was significantly reduced in both groups during the first postoperative decade, similar to that of the general population during the second postoperative decade, and was significantly increased thereafter (Table 2). The RR figures did not differ significantly between groups in any follow-up category (Table 2).

**Table 1:** Number of patients (n) with metal-on-metal or metal-on-polyethylene prostheses stratified by age at operation and number of person-years at risk up to the end of 2005 by age at follow-up.

Age (years)	Metal-on-metal prosthesis		Metal-on-polyethylene prosthesis	
	
	n	Person-years	n	Person-years
20 to 29	2	15.5	4	7.1

30 to 39	6	28.7	16	107.0

40 to 49	25	176.2	55	354.7

50 to 59	145	855.6	291	1800.0

60 to 69	278	2836.3	779	6694.5

70 to 79	113	3898.4	419	10848.2

≥ 80	10	2374.3	21	6595.5

Total	579	10185.1	1585	26406.9

**Table 2:** Observed number of deaths (Obs) (all causes) and standardised mortality ratio (SMR) and rate ratio (RR) among patients with metal-on-metal and metal-on-polyethylene total hip prostheses for osteoarthritis by number of postoperative years.

Follow-up time, completed years	Metal-on-metal prosthesis (n = 579)			Metal-on-polyethylene prosthesis (n = 1585)				
	**Obs**	**SMR**	**95% CI‡**	**Obs**	**SMR**	**95% CI**	**RR**	**95% CI**

0 to 1	16	0.59	0.34-0.96*	35	0.37	0.26-0.51***	1.61	0.83-2.98

2 through 9	122	0.77	0.64-0.91**	342	0.70	0.63-0.78***	1.09	0.88-1.35

10 through 19	200	0.94	0.81-1.06	611	0.96	0.89-1.04	0.97	0.82-1.14

20+	190	1.20	1.04-1.37 *	311	1.38	1.23-1.53***	0.87	0.72-1.05

Total	528	0.95	0.87-1.02	1299	0.90	0.85-0.95***	1.05	0.95-1.16

‡ 95% CI, 95% confidence interval

*p < 0.05, **p < 0.01, *** p < 0.001

Cancer mortality in the MM group was similar to that in the general population, but the rate was significantly reduced in the MP group (Table 3, 4). During the first 20 years after THA, the SMR for the MM group (1.01) was higher than that for the MP group (0.74); RR 1.36 (95% CI 1.02-1.79). After 20 years, however, there was no practical difference between groups (Table 4).

**Table 3:** Observed number of deaths (Obs) and standardised mortality ratio (SMR) among patients with metal-on-metal and metal-on-polyethylene total hip prostheses for osteoarthritis in the main disease groups.

Cause of death	Metal-on-metal prosthesis (n = 579)			Metal-on-polyethylene prosthesis (n = 1585)		
	**Obs**	**SMR**	**95% CI‡**	**Obs**	**SMR**	**95% CI**

Cancer	96	0.97	0.79-1.18	206	0.76	0.66-0.86 ***

Cardiovascular	300	0.96	0.86-1.07	702	0.90	0.84-0.97 **

Respiratory	33	0.67	0.46-0.93 *	101	0.78	0.64-0.94 **

Pneumonia	28	0.87	0.58-1.25	79	0.96	0.76-1.20

Alzheimer, dementia	28	0.96	0.64-1.38	105	1.20	0.98-1.43

Genitourinary	8	0.90	0.39-1.76	27	1.32	0.87-1.91

Digestive system	18	1.08	0.64-1.71	45	1.07	0.78-1.43

Diabetes	6	0.80	0.29-1.74	7	0.42	0.17-0.86*

Accidents and violence	14	0.78	0.42-1.30	38	0.79	0.56-1.07

All causes	528	0.95	0.87-1.02	1299	0.90	0.85-0.95 ***

‡ 95% CI, 95% confidence interval

*p < 0.05, **p < 0.01, *** p < 0.001

**Table 4:** Observed number of cancer deaths (Obs) and standardised mortality ratio (SMR) and rate ratio (RR) with 95% confidence intervals of patients with metal-on-metal and metal-on-polyethylene total hip prostheses stratified by number of postoperative years.

Follow-up time, completed years	Metal-on-metal prosthesis (n = 579)			Metal-on-polyethylene prosthesis (n = 1585)				
	**Obs**	**SMR**	**95% CI‡**	**Obs**	**SMR**	**95% CI**	**RR**	**95% CI**

0 to 1	2	0.35	0.04-1.27	3	0.14	0.03-0.41***	2.51	0.21 -21.94

2 through 9	29	0.92	0.62-1.32	77	0.78	0.61-0.96*	1.19	0.74 - 1.85

10 through 19	43	1.19	0.86-1.59	89	0.84	0.67-1.02	1.42	0.96 - 2.07

20+	19	0.84	0.50-1.30	33	0.89	0.61-1.24	0.95	0.51 - 1.71

Total	93	0.97	0.78-1.18	202	0.76	0.66-0.87***	1.27	0.98 - 1.63

‡ 95% CI, 95% confidence interval

*p < 0.05, **p < 0.01, *** p < 0.001

Cardiovascular diseases dominated as the cause of mortality in both groups; 57% of the MM group and 54% of the MP group died from cardiovascular disease. All-over cardiovascular mortality was low during the first decade postoperatively, equivalent during the next decade, and slightly increased after 20 years postoperatively (Table 5). After 20 years, the SMR was 1.60 (95% CI 1.32-1.92) for women in the MP group compared to 1.45 (95%CI 1.16-1.78) for women in the MM group. These figures were slightly lower for men; 1.09 for men in the MP group (95% CI 0.80-1.44) and 1.26 for men in the MM group (95% CI 0.82-1.84). The increase in mortality after 20 postoperative years was observed for all forms of vascular disease, including cerebrovascular diseases for which the SMRs were 1.56 (95% CI 1.07 - 2.18) in the MM group and 1.22 (95% CI 0.84 -1.96) in the MP group.

**Table 5:** Observed number of cardiovascular deaths (Obs) and standardised mortality ratio (SMR) and rate ratio (RR) with 95% confidence intervals of patients with metal-on-metal and metal-on-polyethylene total hip prostheses stratified by number of postoperative years.

Follow-up time, completed years	Metal-on-metal prosthesis (n = 579)			Metal-on-polyethylene prosthesis (n = 1585)				
	**Obs**	**SMR**	**95% CI‡**	**Obs**	**SMR**	**95% CI**	**RR**	**95% CI**

0-1	7	0.46	0.04-1.27	27	0.50	0.03-0.41***	0.91	0.98 - 1.163

2-9	75	0.81	0.62-1.32	193	0.70	0.61-0.96*	1.16	0.88 - 1.52

10-19	103	0.85	0.86-1.59	326	0.97	0.67-1.02	0.87	0.69 - 1.10

20+	115	1.40	0.50-1.30	156	1.40	0.61-1.24	1.00	0.78 - 1.28

Total	300	0.96	0.78-1.18	702	0.90	0.66-0.87 ***	1.07	0.93 - 1.22

‡ 95% CI, 95% confidence interval

*p < 0.05, **p < 0.01, *** p < 0.001

Diabetic mortality was low in the MP group and slightly decreased in the MM group, but the number of patients was small (Table 3). There was a slightly increased risk for dementia or Alzheimer's disease in the MP group (1.20), which increased with an increase in the time after surgery. After 20 years postoperatively, there were 37 recorded cases with an SMR of 1.54 (95% CI 1.08 - 2.12). In the MM group, the total risk for this disease was close to that of the normal population (0.96) (Table 3). After 20 years, 22 patients in the MM group died from this disease; the SMR was 1.29 (95% CI 0.81 - 1.29).

Mortality from respiratory diseases was low in both groups (Table 3). The SMR for the MM group remained low during the first two decades (0.53) and increased slightly thereafter (0.76). On the other hand, the SMR for the MP group increased from 0.66 to 1.34 during the same time period.

Mortality from genitourinary diseases was slightly decreased in the MM group and slightly increased in the MP group (Table 3). After 20 years, the SMR for these diseases in the MM group was 0.77 and 2.13 in the MP group, but the number of patients was too small to demonstrate statistical significance.

Accidental deaths in both groups were slightly and equally reduced. In patients over 80 years of age, however, accidental death due to falling was significantly reduced in the MP group (SMR 0.45; observed 8, expected 17.72, 95% CI 0.19- 0.88) compared to that in the MM group (0.87).

## Discussion and Conclusions

Total hip arthroplasty dramatically improves a patient's quality of life, but exposes patients to chronic stress from cobalt and chromium ions and particles. The MM THA group included only patients implanted with the first-generation MM design. The material of the McKee-Farrar hip prosthesis was a cast cobalt-chromium-molybdenum alloy, with a carbon content of approximately 0.2% that underwent subsequent heat treatment. Its metallurgical, tribological, and structural properties are inferior to those of modern MM bearings with an average annual wear rate of the femoral heads ranging from 3.3 to 6.6 μm and that of cups from 2.1 to 4.9 μm [24]. Due to the high wear rate, we can assume that if excessive metal ion loading from the McKee-Farrar components did not increase the risk of mortality within this cohort, such risk would not be obvious in recipients of MM implants with improved wear characteristics.

All previous reports of the mortality of THA patients studied a period of up to only 10 years after surgery. According to the results of these studies, the 10-year life expectancy of THA patients is better than that of the general population [25,26], which we also observed in the present study. Patient selection, i.e., the 'healthy patient effect', probably explains a major part of the better survival, but the reason for the duration of this effect in different disease groups is not known. In a Swedish Knee Arthroplasty Register study [27], patients with implantation of knee prostheses for OA had a reduced overall mortality during the first 12 postoperative years, which then increased and became significantly higher than that of the general population. Cardiovascular, gastrointestinal, and genitourinary diseases were the main causes for the increased mortality. The authors suspected a link between the early onset of knee OA and increased mortality. Another study reported reduced SMRs in THA patients aged 65 years and over in the United Kingdom, and an increased mortality rate in younger patients; the preoperative indications, however, were not reported [28].

Total mortality in the present study did not differ significantly between the MM and MP groups across the postoperative decades. After 20 years postoperatively, both groups showed significantly increased mortality due to cardiovascular disease. Altered cardiac function is reported among hard metal workers. A prolonged left ventricular relaxation time without clinical consequences was observed in a series of 203 Finnish cobalt production workers [29]. The authors concluded that cobalt accumulation in the myocardium could affect its function. An estimated daily intake of 6 to 8 mg of cobalt chloride or cobalt sulfate induces cardiomyopathy in heavy beer drinkers when cobalt is used as a foam stabilising agent in the beer [30]. Cardiac function, specifically in terms of cobalt or chromium concentration in the myocardial cells, in THA patients after long-term use of MM or MP prostheses has not been studied. Frustaci et al. [31] observed a large increase in the levels of different trace elements in the myocardium of 13 patients with idiopathic dilated cardiomyopathy, with a 13-fold increase in the concentration of cobalt and a 4-fold increase in the concentration of chromium compared to controls. Relationship of the elevated cobalt and chromium concentrations in the serum of THA patients and cardiomyopathy requires further studies.

In a large cohort of Finnish OA patients with an MP hip prosthesis that was not included among patients in the present study with 153 000 person-years and a mean follow-up of 6.2 years, the SMR for all-site cancer was reduced to 0.54 [32], and in the present series, after a mean of 16.2 years, it was still significantly reduced (0.76). According to a meta-analysis of six Nordic cohorts operated on for OA, covering 374 000 person-years and a mean follow-up of 7.6 years, the standardized incidence rate (SIR) was significantly reduced (SIR 0.93, 95% CI 0.91-0.95) [33]. In another meta-analysis [34] of patients with 1.1 million person-years and a mean follow-up time of 6.2 years operated on for all indications, the SIR was close to unity, 0.98, 95% CI 0.98-0.99). Goldachre et al. [35] also reported an SIR of 0.98, 95% CI 0.94-1.01) among 25 047 THA patients from the United Kingdom with a mean follow-up of 7.7 years. OA itself is also associated with a low comorbidity of cancer. Thomas et al. [36] reported a low SIR of 0.85 (95% CI 0.81-0.88) for men and 0.83 (95% CI 0.80-0.86) for women among patients with all forms of OA in a Scottish cohort. It seems that first-generation MM or MP prostheses do not expose patients to an increased risk of cancer. Cobalt and chromium are essential trace elements and hence a DNA repair system may exist in the body to rectify DNA damage caused by these ions. Nucleotide excision repair effectively removes chromium from DNA adducts [37]. In addition, macrophages detoxify the genotoxic and cytotoxic effects of CoCr particles *in vitro *[38].

Although cancer genesis may thus be controlled by these mechanisms, the long-term stress caused by cobalt and chromium ions may eventually exhaust these functions, which may lead to cellular senescence and apoptosis and eventually to a functional failure of the affected organs. Such development may be reflected by the mortality from other causes.

The number of patients that died from gastrointestinal and urogenital diseases in the present series was too small to determine the influence of the implantation on mortality 20 years postoperatively. The significant increase in mortality from dementia or Alzheimer disease 20 years after the MP group is noteworthy. Scientific evidence of a relationship between cobalt or chromium exposure and this disease, however, is lacking.

A limitation of this study is the relatively small number of patients and therefore chance may explain some of the results. Both groups showed similar development of all site mortality, however, over a long follow-up period. In addition, revisions or bilateral operations were not reported, but both groups were certainly exposed to cobalt and chromium debris due to the degradation of nano-sized metal particles. To our knowledge, revision was usually performed with the insertion of a new MM or MP prosthesis.

Over 90% of the patients MM group in the present series had died. Therefore, their causes of death will not change. Older cells are less tolerant than younger cells to cobalt-chromium particles. Nanometer-sized cobalt-chromium particles induce a greater loss of viability in older human fibroblast in vitro than in cells from younger patients [39]. After the 20^th ^post-operative year, both the MM and MP hip prosthesis group had significantly increased mortality. Thus, even longer follow-up times are needed to estimate the risk of death in patients with modern MM bearings.

## Competing interests

The authors declare that they have no competing interests.

## Authors' contributions

TV: study design, preparation of the manuscript; HB initiation of the study, revision of the manuscript; PPu: statistical analysis, revision of the manuscript; PPa study design, revision of the manuscript; EP: data collection (Finnish Cancer Registry), revision of the manuscript. All authors read and approved the manuscript.

## Pre-publication history

The pre-publication history for this paper can be accessed here:

http://www.biomedcentral.com/1471-2474/11/78/prepub
